# Supplementary material for: Medical Students’ Attitudes Toward AI in Medicine and their Expectations for Medical Education
Source: J Med Educ Curric Dev. 2023 Dec 6;10:23821205231219346. doi: 10.1177/23821205231219346 (PMC10704950; doi:10.1177/23821205231219346)
Supplement: sj-docx-1-mde-10.1177_23821205231219346 - Supplemental material for Medical Students’ Attitudes Toward AI in Medicine and their Expectations for Medical Education [file sj-docx-1-mde-10.1177_23821205231219346.docx]

**Appendix A**

*Perceived reliability:*

- “The AI always provides the advice I require to make my decision.”
- “The AI performs reliably.”
- “The AI responds the same way under the same conditions at different times.”
- “I can rely on the AI to function properly.”
- “The AI analyzes problems consistently.”

*Perceived technical competence:*

- “AI uses appropriate methods to reach decisions.”
- “The AI has sound knowledge about medical problems.”
- “The advice the AI produces is as good as that which a highly competent person could produce.”
- “The AI correctly uses the information I enter.”
- “The AI makes use of all the knowledge and information available to it to produce its solution to the problem.”

*Faith:*

- “I believe advice from the AI even when I don’t know for certain that it is correct.”
- “When I am uncertain about a decision, I believe the AI rather than myself.”
- “If I’m not sure about a decision, I have faith that the system will provide the best solution.”
- “When the AI gives unusual advice, I am confident that it is correct.”

*Perceived trustworthiness:*

- “The AI is deceptive.”
- “The AI behaves in an underhanded manner.”
- “I am suspicious of the system’s intent, action, or outputs.”
- “I am wary of the AI.”
- “The AI’s actions will have a harmful or injurious outcome.”
- “I am confident in the AI.”
- “The AI provides security.”
- “The AI has integrity.”
- “The AI is dependable”
- “The AI is reliable.”
- “I can trust in the AI.”
- “I am familiar with the AI.”

*Perceived intelligence:*

To me AI in medicine seems to be....

- incompetent – competent
- ignorant – knowledgeable
- unintelligent – intelligent
- irresponsible – responsible
- foolish – sensible

*Anthropomorphism:*

To me AI in medicine seems to be....

- fake – natural
- machinelike – humanlike
- unconscious – conscious
- artificial – lifelike
